# Shifts in the Midgut/Pyloric Microbiota Composition within a Honey Bee Apiary throughout a Season

**DOI:** 10.1264/jsme2.ME15019

**Published:** 2015-09-01

**Authors:** Jane Ludvigsen, Anbjørg Rangberg, Ekaterina Avershina, Monika Sekelja, Claus Kreibich, Gro Amdam, Knut Rudi

**Affiliations:** 1 Norwegian University of Life Sciences, Chemistry, Biotechnology and Food science department, Campus Ås, Ås 1432, Norway; 2 Genetic AnalysisP.O. Box 4239, Nydalen, 0401 Oslo, Norway; 3 School of Life Sciences, Arizona State University, P.O. Box 874501, 427 East Tyler Mall, Tempe, AZ 85287, USA

**Keywords:** honey bee workers, midgut/pyloric microbiota, seasonal changes, *Gilliamella apicola*, *Snodgrassella alvi*

## Abstract

Honey bees (*Apis mellifera*) are prominent crop pollinators and are, thus, important for effective food production. The honey bee gut microbiota is mainly host specific, with only a few species being shared with other insects. It currently remains unclear how environmental/dietary conditions affect the microbiota within a honey bee population over time. Therefore, the aim of the present study was to characterize the composition of the midgut/pyloric microbiota of a honey bee apiary throughout a season. The rationale for investigating the midgut/pyloric microbiota is its dynamic nature. Monthly sampling of a demographic homogenous population of bees was performed between May and October, with concordant recording of the honey bee diet. Mixed Sanger-and Illumina 16S rRNA gene sequencing in combination with a quantitative PCR analysis were used to determine the bacterial composition. A marked increase in α-diversity was detected between May and June. Furthermore, we found that four distinct phylotypes belonging to the *Proteobacteria* dominated the microbiota, and these displayed major shifts throughout the season. *Gilliamella apicola* dominated the composition early on, and *Snodgrassella alvi* began to dominate when the other bacteria declined to an absolute low in October. *In vitro* co-culturing revealed that *G. apicola* suppressed *S. alvi*. No shift was detected in the composition of the microbiota under stable environment/dietary conditions between November and February. Therefore, environmental/dietary changes may trigger the shifts observed in the honey bee midgut/pyloric microbiota throughout a season.

Honey bees (*Apis mellifera*) are important crop pollinators and are widely used around the world in agriculture and food production (51). The honey bee is a social insect that harbors a core gut microbiota of eight abundant phylotypes, which accounts for ~95% of all gut bacteria (34, 37). The distinct and relatively simple gut microbiota is transmitted from adult to newly hatched bees through feeding and secretion inside the colony (29). The honey bee gut microbiota is distributed throughout the entire digestive tract, in which the midgut harbors approximately 1–4% and the ileum/rectum over 90% of the most dominant bacteria found in honey bees (35). Several studies have shown the importance of gut symbionts in bee health and disease (20, 22, 28, 52, 53). In honey bees, the digestion of nutrients takes place in the midgut (10, 11, 13), and is aided by gut associated microbes. A recent study reported that the honey bee gut symbiont *G. apicola* degraded pectin (19), a main component of pollen, which is difficult to break down by the host (11).

Worker honey bees are functionally sterile helpers that perform within-nest tasks and forage. Foraging workers encounter different plants and flowers during the season, and bring back nectar and pollen to the colony. In Norway, worker honey bees actively forage between April and September. Colony food intake is a function of local environmental conditions, including temperature and precipitation. Foragers visit the plants that are available at that time of the year, and the resulting pollen and nectar is consumed or stored by the colony (13, 23, 40, 43). In September, beekeepers feed the bees a sugar mix as a replacement for the honey that is harvested. Bees cluster inside the hive between September and April due to the cold weather conditions in Norway (43) (www.norges-birokterlag.no). Honey bee colonies are active in thermo-regulation throughout the Nordic winter, and maintain core temperatures of approximately 20°C, in contrast to an in-hive temperature of approximately 30°C during the foraging season (www.norges-birokterlag.no and www.stadevægt.dk). Energy for heat production comes from the consumption of stored sugars as their winter diet. In the spring, the colony has a high demand for protein due to increased levels of brood rearing. Pollen is the primary amino acid and lipid source for honey bees, and pollen foraging is required for rebuilding colony strength through the production of new bees during the springtime (13). In addition to amino acids and lipids, pollen provides vitamins and minerals, whereas nectar is the primary carbohydrate source for the colony.

Throughout the foraging season, honey bees acquire a transient set of gut bacteria that are horizontally transmitted from the environment surrounding the colony (2). Previous studies have suggested differences in the gut microbiota composition between colonies at different locations, between colonies at the same location, and between individual bees within a colony (16, 17, 26, 34, 37). Another recent study by Corby-Harris, *et al.*(9), which performed repeated sampling of foragers, did not find any significant differences in the core gut microbiota composition at two different points in time (fall and spring) at one specific location. Therefore, some findings indicate a highly variable gut microbiota in honey bees, whereas others suggest a more stable gut microbiota. This potential disagreement warrants further studies.

It currently remains unclear how the microbiota of the honey bee gut responds to environmental fluctuations and dietary changes throughout a season. The gut is also spatially complex. Previous studies established that the honey bee ileum contains a stable microbiota (35, 41), while the midgut and pylorus is highly dynamic due to its peritrophic membrane and refluxes from the ileum (48). We hypothesized that environmental/dietary changes have a considerable impact on the microbiota in this dynamic part of the honey bee gut. As a first pass to address this hypothesis, we examined microbial changes in samples of the midgut/pylorus obtained from bees living in commercial hives during a season. At our location, commercial hives are subject to marked changes in diet and environment. Samples from the active foraging season were compared to the microbiota of clustering bees living in a stable winter environment at the same location. We used high throughput 16S rRNA gene sequencing (*i.e.*, both mixed Sanger- and Illumina sequencing) in combination with quantitative PCR (qPCR) to determine the microbiota composition of the samples. In addition, we performed culturing and *in vitro* competition experiments in order to address questions regarding the biology of select midgut/pyloric bacteria. The results obtained suggest that diet/environment is important for shaping the midgut/pyloric microbiota composition, and contribute to its dynamic nature.

## Materials and Methods

### Bee sampling

In order to perform the bee midgut/pyloric microbiota analysis throughout a foraging season, bees were sampled between May and October 2012 at the Norwegian University of Life Sciences, Ås, Norway. Information regarding available nutrition for foraging bees is summarized in Table 1. Ten worker bees from three separate colonies, for a total of 30 bees per month, were collected. The bees were picked at random with tweezers from three of the ten removable combs in the brood chamber box of each colony (one comb in the front, one in the middle, and one at the back). This sampling method had the following benefits: i) it was easy to repeat between colonies, and ii) it ensured that the bees collected were unlikely to fall into the same behavioral group. Therefore, we obtained samples that were similar between colonies and represented the diverse worker populations of those hives (13). The ten bees from the three different colonies were sampled together, randomized, and then analyzed, and the results were averaged across hives, thereby giving data that represented the biological gut bacterial composition in a population at one location. This was performed for all sampling times, except September, in which samples from different colonies were analyzed separately to obtain a snapshot of possible colony differences.

An additional 30 bees were collected (ten from each of the three colonies) to facilitate the culturing of midgut/pyloric bacteria. Moreover, ten bees (three bees from two colonies and four from one colony) were separately collected and used as an average sample (hereafter referred to as the average July sample) in Illumina MiSeq sequencing, analyzing the 16S rRNA microbiota composition, as well as a control for the DNA extraction procedure’s technical variation. All bees were collected in July 2012 from the same three colonies as those used in the microbiota analysis seasonal study.

In order to analyze midgut/pyloric microbiota under stable nutritional conditions, worker bees were collected from two colonies in November 2011 (24 bees) and February 2012 (30 bees), which had been fed a commercial sugar mix (37% sucrose, 19% glucose, 19% fructose and 25% water, Nordic Sugar A/S, Denmark) for one month and four months, respectively. These colonies were restricted to their hive due to cold weather, and, thus, their environment was stable/constant throughout the sampling period.

### Midgut isolation and DNA extraction

Bees were anesthetized on ice directly after sampling (1), and washed in 50% ethanol before dissection. The whole gut was dissected out using a sterile dissecting forceps, and the stinger was pulled out as the bee was held by the head. By pulling the stinger, the intact digestive tract followed, separating the midgut from the crop, which remained in the bee. An illustration of the dissecting procedure is shown in Fig. S1. The midgut/pylorus was collected in micro tubes (Sarstedt, Germany) containing 0.2 g <106 μm acid-washed glass beads (Sigma-Aldrich, USA) and 500 μL S.T.A.R buffer (Roche, Switzerland) by making a cut with a sterile dissecting scissor within the pylorus part of the digestive tract (Fig. S1).

Mechanical lysis was performed using MagNA Lyzer (Roche); 6,500 rpm for 20 s for 2×; 1 min cooling at 4°C between runs. An automated DNA magnetic bead-based extraction method was used on all samples (developed by Genetic Analysis; http://www.genet-analysis.com). A Quant-iT PicoGreen dsDNA assay (Life Technologies, USA) was used for quantification of the extracted DNA (45).

### Microbiota analyzes

#### Mixed sequencing

PCR was used to amplify approximately 450 bases of bacterial 16S rRNA genes in each midgut/pylorus sample using universal 16S rRNA primers; Forward-F11 5′-TCCTACGGGAGGCAGCAGT-3′, Reverse-A01 5′-GGACTACCAGGGTATCTAATCCTGTT-3′ as previously described (38). PCR was performed with HOT FIREpol DNA polymerase (Solis BioDyne, Estonia) in a final volume of 25 μL. Cycling conditions for PCR: Activation 95°C for 15 min, and 30 cycles of 95°C for 30 s, 60°C for 30 s, 72°C for 60 s, Final elongation 72°C for 7 min. A Quant-iT PicoGreen dsDNA assay (Life Technologies) was used to confirm successful PCR amplification, and the correct amplicon size was confirmed on 1% agarose gel.

Sanger sequencing was performed using the BigDye Terminator v1.1 Cycle Sequencing Kit (Life Technologies) on Exo1-treated PCR products in a total volume of 10 μL. Agencourt CleanSEQ Dye-terminator Removal (Beckman Coulter, USA) was used to purify the end-labeled sequences, and all Sanger sequencing was performed at Hedmark University College on a 3130 xl Genetic analyzer (Life Technologies).

The mixed Sanger sequencing method was first applied by Trosvik, *et al.*(49), and, when used in combination with multivariate curve resolution with an alternating least squares analysis (MCR-ALS) (56), mixed bacteria communities can be analyzed (3, 44). The method first aligned all sequence spectra. The co-occurrence of the different parts of the spectra were then determined with the co-occurring nucleotides that were displayed as the component sequence. During the MCR-ALS analysis, a quality filtering of sequence data was performed, and sequences with low quality were removed. The bacterial components were subsequently base-called, and taxonomy assigned using the Basic Local Alignment Search Tool (BLAST) (http://blast.ncbi.nlm.nih.gov). The relative ratio of the dominant bacterial components in each sample was calculated in the MCR-ALS analysis without assuming closure (not adding up to 100% due to residual noise in each sample). Matlab (MathWorks, USA) was used to determine the mean α-diversity (modified Simpson index). The α-diversity, as explained previously (3), was calculated from the raw aligned spectra from each individual sample and based on the ratio of nucleotide mixing at each position in the sequences. The α-diversity calculation method used in this study was unique to the mixed sequencing approach, in which the values could not be directly compared across different methods.

#### Quantitative PCR

The relative quantity of 16S rRNA genes (bacterial load) was determined through ratio calculations between universal 16S rRNA genes and the honey bee specific vitellogenin gene, both retrieved from qPCR. The vitellogenin primers amplified 150 bp of the vitellogenin gene (DNA), which encodes a yolk precursor protein abundant in blood (1). Both qPCR reactions were performed on the LightCycler 480 II (Roche), and the raw data were imported into the LinReg PCR program for CT values and PCR efficiency calculations (42). The log relative bacteria/bee DNA ratio was calculated (30, 46) using the following formula: =(log[efficiency Bee]×CT Bee)−(log[efficiency Bacteria]×CT Bacteria).

EvaGreen (25, 33), as a fluorescent marker, was used for the vitellogenin qPCR with 5× HOT FIREPol EvaGreen qPCR Mix Plus (Solis Bio Dyne) at a final concentration of 1×. One microliter of diluted gDNA (1:2) was added to the working solution for a final volume of 20 μL. The determination of the dilution factor for qPCR was based on a dilution series experiment conducted prior to the analysis. Cycling conditions: Activation 95°C for 15 min, 40 cycles of; 95°C for 30 s, 54°C for 45 s, 72°C for 30 s. Primers for vitellogenin: Forward 5′-GTTGGAGAGCAACATGCAGA-3′, Reverse 5′-TCGATCCATTCCTTGATGGT-3′ were used (1). A high-resolution melting (HRM) analysis was performed to confirm the specificity of the PCR primers, and a positive control (Bee brain-DNA) and negative control (Nuclease-free water) were added to each run. DNA from the honey bee brain was extracted from one sterile dissected bee brain using the DNeasy Blood and Tissue kit (Qiagen, USA), and its concentration was measured using an ND-1000 spectrophotometer (Thermo Scientific, USA). Universal 16S rRNA qPCR was performed using the TaqMan probe as a fluorescent marker (38) and a positive control (*Lactobacillus kunkeii*) and negative control (Nuclease-free water) were added to each run. The same primer pair as that used for mixed sequencing was applied, and we used 5× HOT FIREPol Probe qPCR Mix Plus (Solis BioDyne) in a 1× concentration, with 1 μL of diluted gDNA (1:2) at a final volume of 20 μL, with the following cycling: Activation 95°C for 15 min and 40 cycles of 95°C for 30 s, 60°C for 60 s.

#### Illumina sequencing

The sample used for this analysis was collected in July 2012 (hereafter referred to as the average July sample), and originated from the same three colonies as those used in the seasonal study. This sample consisted of ten bees, the midgut/pylorus of which were sampled and mixed together into one tube, crushed, and diluted with S.T.A.R buffer. This mixture was then made into aliquots to equal the amount of one sample and represented an average measurement in July 2012. The analysis of this sample was performed in triplicate with tagged-universal PRK primers targeting the 16S rRNA gene (PRK314F and PRK806R) (55), as described in (39), for initial PCR and then pooled before sequencing, after quantification with a Quant-iT PicoGreen dsDNA assay and normalization. The amplicon size was approximately 590 bp and indexing corresponding to the Illumina TruSeq LT set-up was used. Samples were sent to the University of Oslo for 250 bp paired-end MiSeq sequencing (Illumina, USA). The retrieved data were analyzed using the Quantitative Insights Into Microbial Ecology (QIIME) pipeline (6). Regarding OTU classification, the forward reads were quality filtered and clustered at a 99% homology level using a closed-reference *uclust* search against the Greengenes database (15). Additionally, the random selection of 1,000 paired-end reads was assigned taxonomic nomenclature using BLAST with a cut-off of 95% identity.

### Technical controls

A positive extraction control was included in each plate run to address potential extraction procedure bias. We used the average July sample for this purpose. Because the bees throughout the season were collected at different time points, DNA was extracted in three turns on a 96-well plate. DNA from the average July sample was extracted in duplicate for each DNA extraction/plate, and this plate setup was maintained throughout the experiment and then used in the subsequent analysis of mixed sequencing and qPCR analysis. The three extraction plates each included the extraction control sample in two replicates, which then resulted in six measurements for this sample. In addition, a non-template control (elution buffer) was included in each run. Both controls followed the same workflow as the experimental samples, and this procedure ensured the detection of potential methodology bias.

### Bacterium isolation and taxonomy assignment

We sampled a separate set of 30 bees in July 2012 from the same three colonies as those for the seasonal dataset and Illumina sequencing. Ten midguts/pylori were pooled in one micro tube (Sarstedt), and prepared with 500 μL 1× phosphate buffered saline (PBS) and 15% glycerol. The gut parts were frozen at −80°C before culturing. Frozen gut parts from one micro tube were homogenized and spread in parallel on Tryptic soy agar plates (TSA) (Merck KGaA, Germany) with 5% horse blood (hereafter referred to as blood agar plates; ThermoFisher Scientific, USA). The blood agar plates were incubated for two d at 37°C in a CO_2_- enriched atmosphere (GasPack EZ CO_2_ container system; Becton Dickinson [BD], USA) (31). Controls for sterility and the correct atmosphere were included. Colonies were randomly picked and discriminated by different morphologies and then repeatedly streaked on new blood agar plates to ensure pure cultures.

DNA extraction from the bacterial isolates was performed as previously described in this study. Universal bacteria CoverAll primers (developed by Genetic Analysis and publically available through purchase) were used for the 16S rRNA gene amplification (amplicon about 1,200 bp, targeting V3–V9) of the bacteria isolates. We used the HOT FIREpol DNA polymerase as previously described with the following cycling conditions: Activation 95°C for 15 min and 30 cycles of 95°C for 30 s, 55°C for 30 s, 72°C for 1 min and 20 s. Sequencing was performed as previously described. The Sanger sequences were processed with the use of CLC Main work bench 6 (CLCbio, Qiagen). Sequencing was performed using both the forward and reverse CoverAll primers, and consensus sequences were assembled from the two complimentary sequences derived from each bacterium and matched in BLAST. Sequence taxonomy was assigned with hits of more than 99% matches. Nearest identity BLAST hits were chosen as reference sequences for all isolates and the mixed sequencing components, and reference sequences for the Illumina sequencing BLAST search were also added. The sequences were then aligned and manually curated and a neighbor-joining phylogenetic tree (jukes cantor algorithm) with bootstrapping (100) was created in CLC main workbench 6.

### Competition experiment

Two of the isolates (*Gilliamella apicola* and *Snodgrassella alvi*) were selected for a competition experiment to determine whether they exhibited symbiotic, mutualistic, or competitive characteristics when grown together. The two strains were selected after screening the isolates with specific primers to yield positive amplification. The primer pair Gamma1-459-qtF 5′-GTATCTAATAGGTGCATCAA TT-3′ and Gamma1-648-qtR 5′-TCCTCTACAATACTCTAGTT-3′ was used to detect *G. apicola*, while the primer pair Beta-1009-qtF 5′-CTTAGAGATAGGAGAGTG-3′ and Beta-1115-qtR 5′-TAAT GATGGCAACTAATGACAA-3′ was used to detect *S. alvi*, as described previously (35). Both isolates were grown alone for one d in tryptic soy broth (TSB) (Merck KGaA) (31) in 1.5-mL Eppendorf tubes prepared with sterile 0.2 g <106 μm acid-washed glass beads with a starting amount of 1 μL swabbed from a blood agar plate dissolved in 100 μL TSB. All 100 μL were mixed with the respective bacterium and they were then grown together and alone under the same condition as described above for two more d with additional TSB in a total volume of 1.5 mL. The experiment was performed in triplicate, and a negative control (only TSB) was added. The bacteria were lysed, and DNA was extracted for qPCR quantification of the bacterial load in each sample on LightCycler 480 II. qPCR was performed on all samples in one run, in duplicate, using 5× HOT FIREPol EvaGreen qPCR Mix Plus at a final concentration of 1× with the following cycling conditions: Activation 95°C for 15 min and 40 cycles of 95°C for 30 s, 55°C for 30 s, 72°C for 30 s. The products (amplicon size; *G. apicola* 210 bp, *S. alvi* 128 bp) were verified by a HRM analysis and on a 1.5% agarose gel. qPCR raw data were processed using the LinReg PCR program as previously described, and the ratio between the same bacterium grown alone and together with the other bacterium was calculated.

### Statistical analysis

Each point in time consisted of data measurements from single bees that were added and averaged. The standard deviation (SD) and standard error of the mean (SEM) were determined for all points in time. We performed a one-way ANOVA to test the complete seasonal trend for each bacterium. The same statistical method was applied for the qPCR results and α-diversity analysis. We used the Tukey HSD test to test the significant difference among different points in time. In addition, we performed two-sided Student’s *t*-tests for comparisons between bee colonies and to address technical variations. Corrections for multiple testing (Bonferroni) were performed by dividing the selected *p*-value on the numbers of *t*-tests performed in each analysis.

### Accession numbers

Sanger-sequences were deposited in GenBank under accession numbers KM454389–KM454422.

## Results

### Overall microbiota composition

We used Illumina sequencing, mixed sequencing, and culturing to determine the overall composition of the microbiota in our dataset.

Illumina sequencing of the average July sample gave a total of 188,189 reads after quality filtering. QIIME analyses showed low diversity (Fig. S2, Table S1). Unfortunately, most likely due to a lack of bee-associated bacteria in the Greengenes database, we could not obtain accurate taxonomic assignments by QIIME. Therefore, we performed an in-depth BLAST search of 1,000 random Illumina sequences. These analyses showed that the five most abundant bacteria identified were: *Tatumella* sp. 40% (which gave an equal number of hits on both *Tatumella ptyseos* and *Tatumella terrea*), *G. apicola* 23%, *S. alvi* 16%, *Frischella perrara* 12%, and *Lactobacillus kalixensis* 6%.

**Fig. 1:**
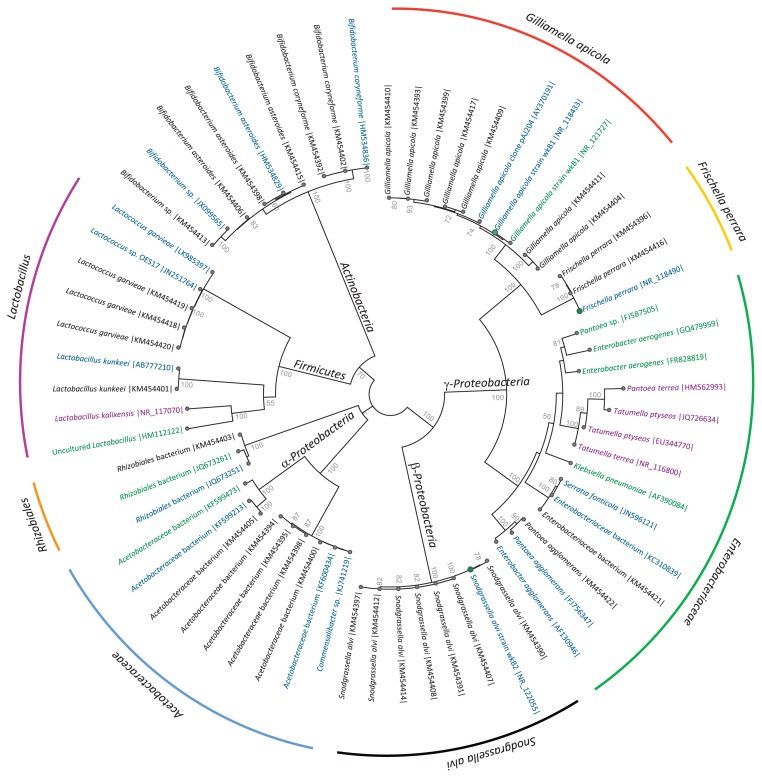
Neighbor-joining phylogenetic tree of bacterial isolates from the honey bee midgut/pylorus. All 34 bacteria cultured and isolated from the honeybee midgut/pylorus collected in July 2012 were included (black text), and their reference sequences (>99% identity BLAST hits) have blue colored text. Reference sequences for Illumina BLAST hits are marked with purple text and blue text with a green node. In addition, the best percent identity BLAST hit sequences for the four bacterial components retrieved from mixed sequencing are included with green colored text. The tree was made using CLC Main workbench 6 and bootstrap values over 50% are shown. The colored circle outside the main tree shows the BLAST based taxonomic assignments for the bacterial components from mixed Sanger sequencing. The following color codes were used; going in a clockwise direction: red; *Gilliamella apicola*, yellow; *Frischella perrara*, green; *Enterobacteriaceae*; black; *Snodgrassella alvi*, blue; *Acetobacteraceae*, orange; *Rhizobiales* bacterium, purple; *Lactobacillus*.

The cultivation of midgut/pylorus samples resulted in 34 bacteria isolates, from five different bacterial phyla, which clustered with previously identified gut bacteria from honey bees (Fig. 1).

Seven main bacterial components were identified by mixed sequencing (Table 2 and Fig. S3). These bacterial components were matched with both Illumina sequencing and cultured isolate sequences, representing most of the characterized diversity (Fig. 1).

Four of these bacterial components were identified in the seasonal dataset and were taxonomically assigned as *F. perrara*, *G. apicola*, *S. alvi*, and one component, which had the closest % identity to the *Enterobacteriaceae* family. Three additional components were identified in the stable environment dataset: *Acetobacteraceae*, *Rhizobiales*, and *Lactobacillus*.

### Seasonal trends in population composition

**Fig. 2:**
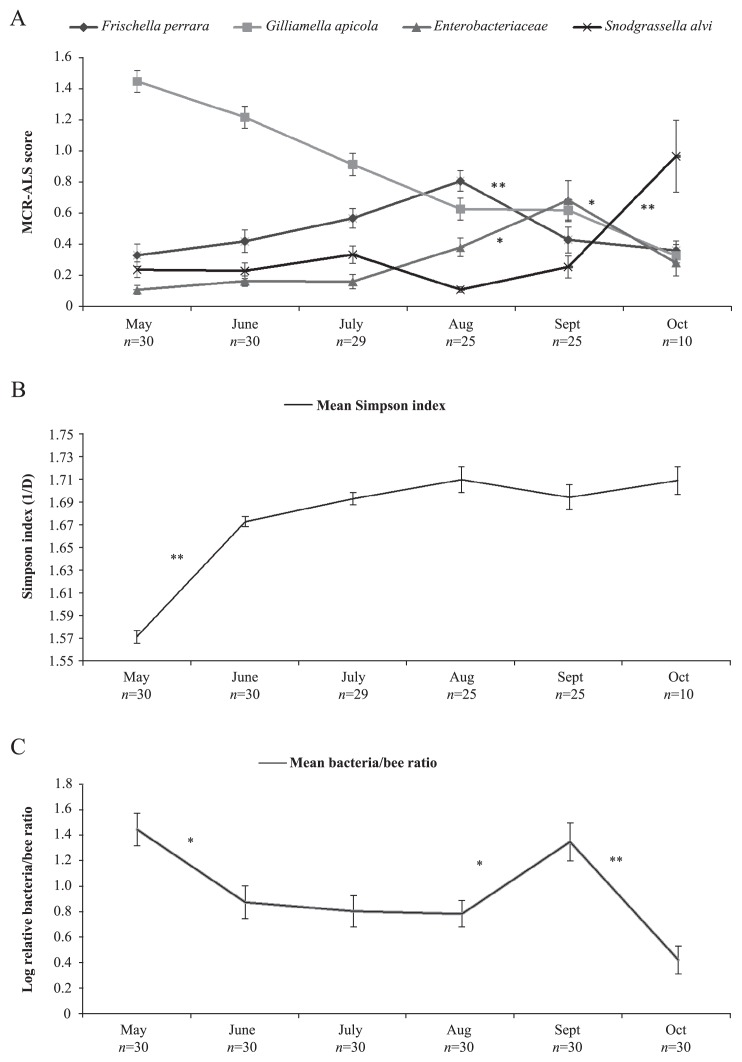
Seasonal changes in the honey bee midgut/pyloric microbiota. A) Mean microbiota composition throughout a foraging season between May and October for the four dominating components found in the honey bee midgut/pylorus by mixed sequencing. The MCR-ALS score, determined by mixed sequencing, represents an approximately relative bacterial composition in the honey bee midgut/pylorus without assuming closure of the system. B) α-diversity between May and October calculated from the raw spectra of mixed sequencing. C) Mean bacterial load for each month between May and October. The calculated relative ratio between 16S rRNA genes and vitellogenin genes (bacteria/bee), in the midgut/pylorus determined by quantitative PCR, is shown. Significant differences were observed between May and October (one-way ANOVA *p*=<0.01) in the three analyses, and *n*; number of bees included in the final analyses each month. Markings show the error bars of the calculated SEM (2α=68.2% CI) for each month and Tukey HSD significant difference values, from pair-wise comparisons of the neighborly time points of the monthly average values of *n* bees, is shown; *=*p*<0.05, **=*p*<0.01.

Major changes in the midgut/pyloric bacterial relative abundance were evident between May and October, and the calculated Tukey HSD test *p*-values gave significance on the 95% and 99% levels (Fig. 2a, Table S2). *G. apicola* showed high dominance early on in the season, but its relative abundance declined between May and August, with the lowest point being reached in October. A significant difference was observed in the relative abundance of *F. perrara* between August and September with a dominance peak in August, and similar results were obtained for *Enterobacteriaceae* between August and October, but with a dominance peak in September. The relative abundance of *S. alvi* was low for the first three months, then declined in August to its lowest point, but increased at the end of the season. An approximately eight-fold increase in the abundance of *S. alvi* was detected between September and October. Calculations on α-diversity gave a markedly lower diversity in May, which significantly increased until July, and then remained fairly stable throughout October (Fig. 2b). The one-way ANOVA for both the four main bacteria and α-diversity displayed significant *p*-values of *p*<0.01 with respect to the temporal trends between May and October (Table S2).

The relative quantity of 16S rRNA genes (bacterial load) was the highest in May and peaked again in September, and the lowest point being reached in October (Fig. 2c). Tukey HSD significant differences between May and June, August and September, and September and October measurements were determined at the 95% and 99% levels (Fig. 2c, Table S2). The one-way ANOVA for the whole dataset revealed significance of *p*<0.01 between May and October (Table S2).

### Colony variation

Colony variations were examined among the three colonies in September, and no significant differences were observed in bacterial relative abundance after a *t*-test Bonferroni correction (Fig. S4A). However, one colony (colony 3) had a significantly higher relative bacterial load (*p*<0.01) than the two other colonies (Fig. S4B).

### Population composition in a stable environment

**Fig. 3:**
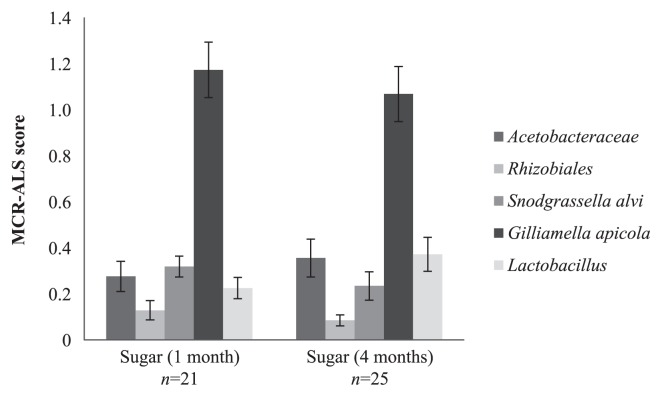
The honey bee midgut/pyloric microbiota composition in the stable environment dataset. The mean microbiota composition at two different time points (November and February) under stable dietary conditions (sugar-mix) for the five dominating components found in the honey bee midgut/pylorus by mixed sequencing. At the two time points, November and February, the bees had been fed the commercial sugar mix for one month and four months, respectively. The MCR-ALS score, determined by mixed sequencing, represents an approximately relative bacterial composition in the honey bee midgut/pylorus without assuming closure of the system. Markings show the error bars of the calculated SEM (2α=68.2% CI) and *n*; number of bees included in the final analyses each month.

The two time point analysis of the two colonies feeding on stored sugars through the winter showed no significant difference after a *t*-test Bonferroni correction either in bacterial relative abundance between November and February (Fig. 3) or in relative bacterial load (bacteria/bee ratio), which was 0.15±0.08 and 0.25±0.1 for November and February, respectively.

### Competition experiment

**Fig. 4:**
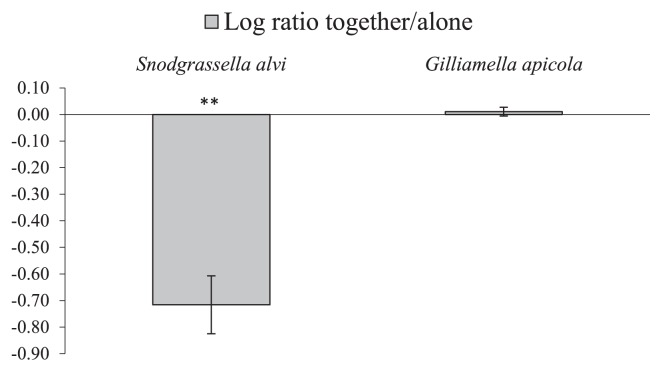
*In vitro* competition experiment with *Gilliamella apicola* and *Snodgrassella alvi*. Significant differences were observed in the relative ratio between *S. alvi* when grow alone and when grown together with *G. apicola*. Markings show the error bars of the calculated SEM (2α=68.2% CI), *t*-test: **p=<0.01. The calculated mean values from three independent experiments are shown.

The results obtained in the seasonal study suggested a negative interaction between the two bacterial species *S. alvi* and *G. apicola*. Therefore, these bacteria were selected for an *in vitro* competition experiment. When grown alone, *G. apicola* and *S. alvi* both showed a steady state bacterial load; however, when grown together, *S. alvi* showed significantly less growth (Fig. 4). In contrast, *S. alvi* did not influence the growth of *G. apicola*; no significant differences were observed from *G. apicola* grown alone.

### Technical validation

The calculated average value of two replicates on each plate indicated minor plate variations in the bacterial relative abundance analysis (Fig. S5). *G. apicola* in this average July sample showed a mean difference of 0.3 units in the MCR-ALS score between May and August, which was the largest difference observed. The qPCR analysis showed a bacterial to bee ratio of 0.59±0.114 with respect to plate-to-plate variations.

Of the 30 midguts/pylori analyzed each month, the resulting number of samples after quality filtering during the MCR-ALS analysis was: May 100%, June 100%, July 97%, August 83%, September 83%, and October 33%. The reduced number of sequences passing quality filtering in October may have been due to the low amount of bacterial DNA that month (as determined by qPCR). Of the 54 bees (24 in November+30 in February) collected for the stable environment dataset, 87% and 83%, respectively, passed the quality filter.

## Discussion

Our approach enabled the detection of distinct shifts in honey bee midgut/pyloric bacteria throughout the season. This was in contrast to Corby-Harris, *et al.*(9), who found no significant difference between the bacterial composition in spring and fall when characterizing the total gut community (crop, midgut, ileum and rectum) of foraging bees. Due to the high amount of bacteria in the hindgut (the lower part of ileum and rectum) (35), the microbiota composition in reference to this part may have been overrepresented if the intestine (midgut, ileum, and rectum) was analyzed as a whole. Therefore, our results support the midgut/pyloric microbiota being more dynamic than the hindgut microbiota. The bacterial composition of the midgut/pylorus may shift because of local metabolic processes, and/or the repeated shedding of the peritrophic membrane and reflux from the ileum (48). At the same time, the highly structured seasonal shifts observed suggest that external forces may play a significant role in shaping the midgut/pyloric microbiota. We favored environmental exposure and dietary changes as the main drivers for seasonal trends because of the strong relationship that exists between gut bacterial composition and the host diet in humans, vertebrate animals, and insects (7, 12, 14, 24). In support of this view, our measurements of the midgut/pylorus microbiota composition from hives fed the same diet over a period of four months showed a stable microbiota composition.

The marked increase observed in α-diversity between May, June, and July may, in some extent, be explained by shifts in the dominating bacteria. Comparisons between mixed sequencing and α-diversity calculations indicated that the decrease in *G. apicola* alone can not completely describe the increase in α-diversity between May and June. Therefore, we also considered this increase to be influenced by the colony being more exposed to various environmental bacteria as well as additional dietary compounds when bees start to forage than when the colony does not forage during the winter (2, 9). Although bees were foraging in May (starting in April in 2012), there was a limited food supply in Ås, Norway at that time of the year, and the available foraging plants provided pollen as a main nutrient. In June, more flowers and plants emerged and persisted, and various nectar and some pollen sources were available. This richer foraging context continued throughout the summer with more nectar-bearing flowers becoming available. We speculated that the high α-diversity that was still prevalent in October indicated that bacteria from the peak foraging season persisted in stored food reserves inside the hive for some time (2).

The peaks in bacterial load appeared to correlate with the two main dietary changes in May and September. The bacterial peak in May most likely reflected the *G. apicola* component, which we found to be highly dominant at this point. Previous studies reported that *G. apicola* was the most abundant in the ileum (35), and is the sole bacterium in the honey bee gut that is able to degrade pectin (19). Pectin is a main constituent of pollen (47), and its degradation is known to occur in the midgut (27). These findings indicate that bees foraging early on in the season mostly acquire pollen as a nutrient; hence, bacteria able to utilize pollen will proliferate and dominate in the midgut/pylorus. The peak in September likely reflected the proliferation of *Enterobacteriaceae* because this component dominates the bacterial composition in September. The start of sugar feeding in September and nectar foraging during August may both have influenced this proliferation. The latter may have had a stronger influence because elevated levels of *Enterobacteriaceae* were already detected in July/August when the bees were still foraging. BLAST hits of our *Enterobacteriaceae* component gave the best percent identity to different bacteria genera previously isolated from honey bee guts and from plants (2, 4, 54). Furthermore, our results were consistent with previous findings by Corby-Harris, *et al.*(9) who detected *Enterobacteriaceae* only in the gut samples of forager bees in fall. Comparisons of this component to both Illumina and culturing BLAST hits gave a span of four different bacterial genera (*Pantoea*, *Enterobacter*, *Tatumella*, and *Serratia*). Reclassification within these genera in recent years, exemplified by *Enterobacter agglomerans* being transferred to *Pantoea agglomerans* by Gavini, *et al.*(21), and *Pantoea* sp. being assigned to *Tatumella* sp. by Brady, *et al.*(5), appeared to connect these results together.

Colony demography has been suggested to play a role in measuring the total bacterial load and bacterial relative abundance throughout a season. The production of new bees by the bee colony is seasonal; one-d-old bees harbor at least three orders of magnitude fewer bacteria than older workers (35). A recent study by Powell, *et al.*(41) showed differences in the microbiota composition between newly hatched bees and 16-d-old bees. We collected ten bees randomly from three different combs resulting in 30 bees per time point. This method ensured a broad representation of ages and task groups; however, since we did not sample by age, we cannot rule out some age-related influence on the gut microbiota. However, Martinson, *et al.*(35) reported that the bacterial load in young workers increased rapidly (within nine d) to that in older bees. Due to this rapid colonization, it is unlikely that our dynamic results were solely driven by changes in colony demography. The result obtained in October, with the high prevalence of *S. alvi*, may have been influenced by age-related differences. Worker bees become more similar in age as the colony prepares for winter because the production of new bees slows down and stops and the oldest bees die out (36). This compression of age in the worker caste may explain the abrupt change observed in the bacterial composition in October because the prevalence of *S. alvi* was high in the ileum of young bees (41).

A separate factor that also needs to be considered is temperature changes, which may have a major impact on bacterial communities. However, although ambient temperatures markedly change throughout the year in Norway, the within-colony environment of honey bees is more stable, with minor changes in core temperatures and only 10–20°C variations at the periphery (8, 40, 43), (www.norges-birokterlag.no, www.stadevægt.dk). In contrast to the changing ambient environment, the midgut/pyloric microbiota remained stable between November and February in our colonies. This stability suggests that the shifts observed in the relative abundance of bacteria were not driven by temperature; however, some influences of temperature cannot be ruled out and require further study.

When we compared our bacterial load results with those from the bacterial relative abundance analysis, we found a two-fold decrease in the total bacterial load in October, corresponding to an approximately four-fold increase in *S. alvi* within the bacterial composition. This, as an additional explanation for the October result, indicated *S. alvi* outgrowth only when there were few other bacteria present. Suppression was confirmed by one strain of *G. apicola* in our *in vitro* competition experiment, which showed that, when competing for the same nutrients and grown under set conditions, *G. apicola* suppressed *S. alvi* outgrowth. Although we cannot generalize from two strains, recent findings support these two species occupying different niches: Martinson *et al.*(35) conducted FISH staining of the honeybee gut and found that *S. alvi* adhered to the midgut/ileum wall, forming a bacterial layer, whereas *G. apicola* habited the luminal niche. *G. apicola* and *S. alvi* occupying different niches is in line with recent evidence for genome complementarity between these two bacteria (32). Furthermore, these two bacteria have been shown to exhibit different growth properties when grown in broth. *S. alvi* without flagella (17) grew in the bottom of a tube as a bacterial layer (31), whereas *G. apicola* with its flagella (17) was suited for competition for nutrients throughout the whole broth (31). We detected similar growth properties for our strains. These findings together with the present results indicate that, *in vivo*, luminal *G. apicola* may prevent *S. alvi* from entering the luminal niche.

The mixed Sanger sequencing used in this study was originally applied and validated for a time series analysis of mixed bacteria communities (50). The limitations of this method lie in the detection of low abundance species in high richness communities. The bacteria, which we discovered dominating the midgut/pyloric microbiota, in our dataset were previously identified as major constituents in the honey bee gut (18, 31, 34, 37), thereby supporting the suitability of the analyses. The relatively simple and defined honey bee gut composition makes mixed Sanger sequencing a better choice than high throughput sequencing because it is cheaper and less computer intensive. In addition, our OTU classification by QIIME could not distinguish between *Frischella* and *Gilliamella*, but rather classified them as *Pasteurellales*. Both bacteria were identified using Sanger sequencing, which indicated the higher sensitivity of Sanger-sequencing reads when identifying highly similar sequences. Mixed sequencing revealed that our *Enterobacteriaceae* component did not dominate the bacterial composition in July, which is in contrast to the results obtained by Illumina sequencing. Therefore, Illumina sequencing may have inferred a bias in these sequences because *Enterobacteriaceae* did not dominate amongst our bacteria isolates (only 2 out of 24 isolates) or in the gut microbiota in other studies.

## Conclusion

The results of the present study exemplify major changes in the honey bee midgut/pyloric microbiota composition throughout a foraging season, whereas a stable microbiota composition was maintained under stable environmental conditions during winter. We emphasize the need for longitudinal studies to investigate and understand the gut microbiota in honey bees.

## Supplementary Information



## Figures and Tables

**Table 1:** Diet of honey bees throughout a foraging season

Sample time point	Diet
May	Dandelion, maple, and fruit trees
June	Raspberries
July	Raspberries & canola
August	Heather & honeydew
September	Sugar mix^1^ (1 week^2^)
October	Sugar mix^1^ (1 month^3^)

1 Sugar mix composition: 37% sucrose, 19% glucose, 19% fructose, and 25% water.

2 Bees had been fed this sugar mix for one week by the sampling time point.

3 Bees had been fed this sugar mix for one month by the sampling time point.

**Table 2:** Assigned taxonomy by BLAST hits for bacterial components derived from mixed sequencing

Component	Assigned taxonomy	Accession No. GenBank	Dataset	E-value	% identity
*Frischella perrara*	*Frischella perrara*	NR_118490	Seasonal	1e-73	96
*Gilliamella apicola*	*Gilliamella apicola*	NR_121727	Seasonal/Stable environment	1e-83	99
*Snodgrassella alvi*	*Snodgrassella alvi*	NR_122055	Seasonal/Stable environment	1e-73	95
*Enterobacteriaceae*	*Klebsiella pneumonia*	AF390084	Seasonal	3e-60	91
	*Pantoea* sp.	FJ587505		3e-59	90
	*Enterobacter aerogenes*	FR828819		3e-59	90
*Acetobacteraceae*	*Acetobacteraceae* bacterium	KF599473	Stable environment	5e-53	89
*Rhizobiales*	*Rhizobiales* bacterium	JQ673261	Stable environment	5e-83	99
*Lactobacillus*	Uncultured *Lactobacillus* sp.	HM112122	Stable environment	2e-47	87
