# Supplementary material for: Shifts in the Midgut/Pyloric Microbiota Composition within a Honey Bee Apiary throughout a Season
Source: Microbes Environ. 2015 Sep 1;30(3):235–44. doi: 10.1264/jsme2.ME15019 (PMC4567562; doi:10.1264/jsme2.ME15019)
Supplement: Supplementary file 1 [file 30_235_s1.pdf]

Table S1: OTU classification after QIIME analysis of Illumina data.

**OTU table**	**Illumina sequencing of midgut/pyloric samples from honey bees in Norway (20 most abundant OTUs)**				**Calculated average**
p__Proteobacteria	c__Gammaproteobacteria	o__Enterobacteriales	f__Enterobacteriaceae	g__Enterobacter	0,381882656
p__Proteobacteria	c__Gammaproteobacteria	o__Pasteurellales	f__	g__	0,374901139
p__Proteobacteria	c__Betaproteobacteria	o__Neisseriales	f__Neisseriaceae	g__	0,092378554
p__Firmicutes	c__Bacilli	o__Lactobacillales	f__Lactobacillaceae	g__Lactobacillus	0,052940745
p__Proteobacteria	c__Alphaproteobacteria	o__Rhodospirillales	f__Acetobacteraceae	g__	0,035502138
p__Proteobacteria	c__Gammaproteobacteria	o__Enterobacteriales	f__Enterobacteriaceae	g__	0,027983411
p__Proteobacteria	c__Alphaproteobacteria	o__Rhizobiales	f__Bartonellaceae	g__	0,00724586
p__Actinobacteria	c__Actinobacteria	o__Bifidobacteriales	f__Bifidobacteriaceae	g__Bifidobacterium	0,007081892
p__Proteobacteria	c__Gammaproteobacteria	o__Pseudomonadales	f__Moraxellaceae	g__Acinetobacter	0,004898881
p__Bacteroidetes	c__Flavobacteriia	o__Flavobacteriales	f__Flavobacteriaceae	g__	0,002523648
p__Proteobacteria	c__Gammaproteobacteria	o__Enterobacteriales	f__Enterobacteriaceae	g__Citrobacter	0,002435845
p__Proteobacteria	c__Gammaproteobacteria	o__Aeromonadales	f__	g__	0,001923667
p__Proteobacteria	c__Alphaproteobacteria	o__Rhodospirillales	f__Acetobacteraceae	g__Gluconobacter	0,001701546
p__Cyanobacteria	c__Chloroplast	o__Streptophyta	f__	g__	0,000950962
p__Proteobacteria	c__Betaproteobacteria	o__Burkholderiales	f__Comamonadaceae	g__	0,000377583
p__Proteobacteria	c__Gammaproteobacteria	o__Enterobacteriales	f__Enterobacteriaceae	g__Gluconacetobacter	0,000376757
p__Bacteroidetes	c__Bacteroidia	o__Bacteroidales	f__Bacteroidaceae	g__Bacteroides	0,000328932
p__Proteobacteria	c__Gammaproteobacteria	o__Pseudomonadales	f__Pseudomonadaceae	g__Pseudomonas	0,000316566
p__Proteobacteria	c__Gammaproteobacteria	o__Enterobacteriales	f__Enterobacteriaceae	g__Trabulsiella	0,000281057
p__Proteobacteria	c__Alphaproteobacteria	o__Sphingomonadales	f__Sphingomonadaceae	g__Sphingomonas	0,000267303

Calculated average abundance of triplicates from a pooled sample of ten midguts.

Table S2: Results of ANOVA and TukeyHSD test for each bacterial component from Mixed sequencing, alfa-diversity and relative bacteria/bee ratio per month.

ANOVA One way

	Effect	SS	Degree of I	MF	F-value	P-value
**Bakt/bie ratio**	Months	21.69	5	4.338	9.508	.000*
**Alfa div**	Months	0.527	5	0.105	49.38	0.00*
**Frischella**	Months	3.804	5	0.761	5.774	.000*
**Gilliamella**	Months	18.19	5	3.638	17.56	.000*
**Snodgrassella**	Months	5.555	5	1.111	10.95	.000*
**Enterobacteriaceae**	Months	5.943	5	1.189	10.87	.000*

Tukey HSD test; variable Ratio Bact/ Bee

Cell No.	Months	{1}	{2}	{3}	{4}	{5}	{6}
1	Mai		0.014917	0.003678	0.002314	0.994076	0.000020
2	Juni	0.014917		0.998728	0.995448	0.076006	0.107170
3	Juli	0.003678	0.998728		0.999996	0.024263	0.255230
4	Aug	0.002314	0.995448	0.999996		0.016459	0.318812
5	Sept	0.994076	0.076006	0.024263	0.016459		0.000022
6	Okt	0.000020	0.107170	0.255230	0.318812	0.000022	

Tukey HSD test; variable Simpson index

Cell No.	Months	{1}	{2}	{3}	{4}	{5}	{6}
1	**May**		0.000020	0.000020	0.000020	0.000020	0.000020
2	**June**	0.000020		0.320912	0.011833	0.307235	0.135416
3	**July**	0.000020	0.320912		0.665365	0.999997	0.887062
4	**August**	0.000020	0.011833	0.665365		0.761656	1.000000
5	**September**	0.000020	0.307235	0.999997	0.761656		0.923161
6	**October**	0.000020	0.135416	0.887062	1.000000	0.923161	

Tukey HSD test; variable Gilliamella apicola

Cell No.	May	{1}	{2}	{3}	{4}	{5}	{6}
1	May		0.391108	0.000158	0.000020	0.000020	0.000020
2	June	0.391108		0.112450	0.000049	0.000042	0.000022
3	July	0.000158	0.112450		0.202220	0.178381	0.006690
4	August	0.000020	0.000049	0.202220		1.000000	0.500471
5	September	0.000020	0.000042	0.178381	1.000000		0.529241
6	October	0.000020	0.000022	0.006690	0.500471	0.529241	

Tukey HSD test; variable Frischella perrara

Cell No.	Months	{1}	{2}	{3}	{4}	{5}	{6}
1	May		0.933560	0.124601	0.000040	0.916583	0.999901
2	June	0.933560		0.627225	0.001290	0.999999	0.998030
3	July	0.124601	0.627225		0.157248	0.735202	0.638987
4	August	0.000040	0.001290	0.157248		0.003569	0.014369
5	September	0.916583	0.999999	0.735202	0.003569		0.996300
6	October	0.999901	0.998030	0.638987	0.014369	0.996300	

Tukey HSD test; variable Enterobacteriaceae

Cell No.	May	{1}	{2}	{3}	{4}	{5}	{6}
1	May		0.981278	0.985505	0.026677	0.000020	0.691258
2	June	0.981278		1.000000	0.153481	0.000020	0.930303
3	July	0.985505	1.000000		0.148954	0.000020	0.924390
4	August	0.026677	0.153481	0.148954		0.017282	0.966692
5	September	0.000020	0.000020	0.000020	0.017282		0.015979
6	October	0.691258	0.930303	0.924390	0.966692	0.015979	

Tukey HSD test; variable Snodgrassella alvi

Cell No.	May	{1}	{2}	{3}	{4}	{5}	{6}
1	May		0.999998	0.875005	0.667620	0.999977	0.000020
2	June	0.999998		0.823480	0.722097	0.999789	0.000020
3	July	0.875005	0.823480		0.105036	0.946986	0.000021
4	August	0.667620	0.722097	0.105036		0.590828	0.000020
5	September	0.999977	0.999789	0.946986	0.590828		0.000020
6	October	0.000020	0.000020	0.000021	0.000020	0.000020	

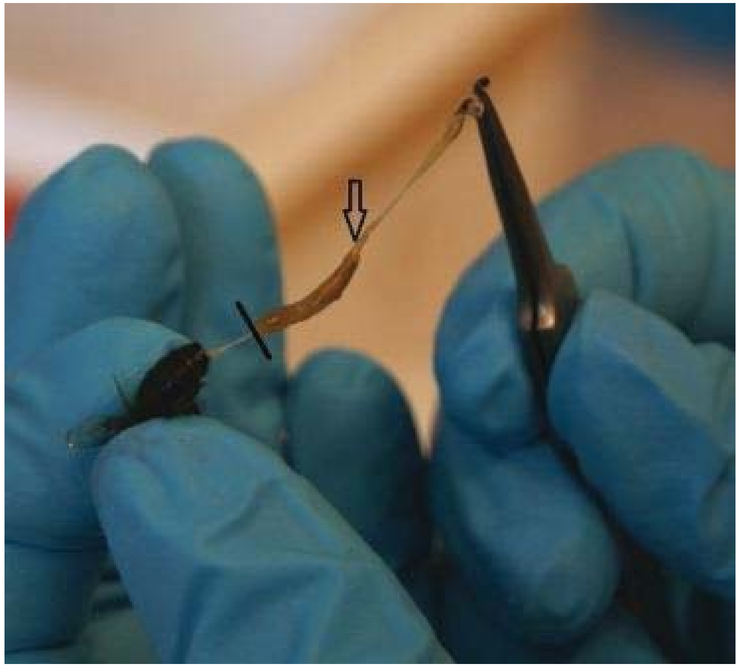

Fig. S1: Midgut/pylorus dissection procedure. The gut was pulled out of the anesthetized bee by the stinger and the midgut/pylorus was collected into sterile container by cutting in the transition between the ileum and the pylorus (arrow). The midgut was separated from the crop by itself when pulling (line).

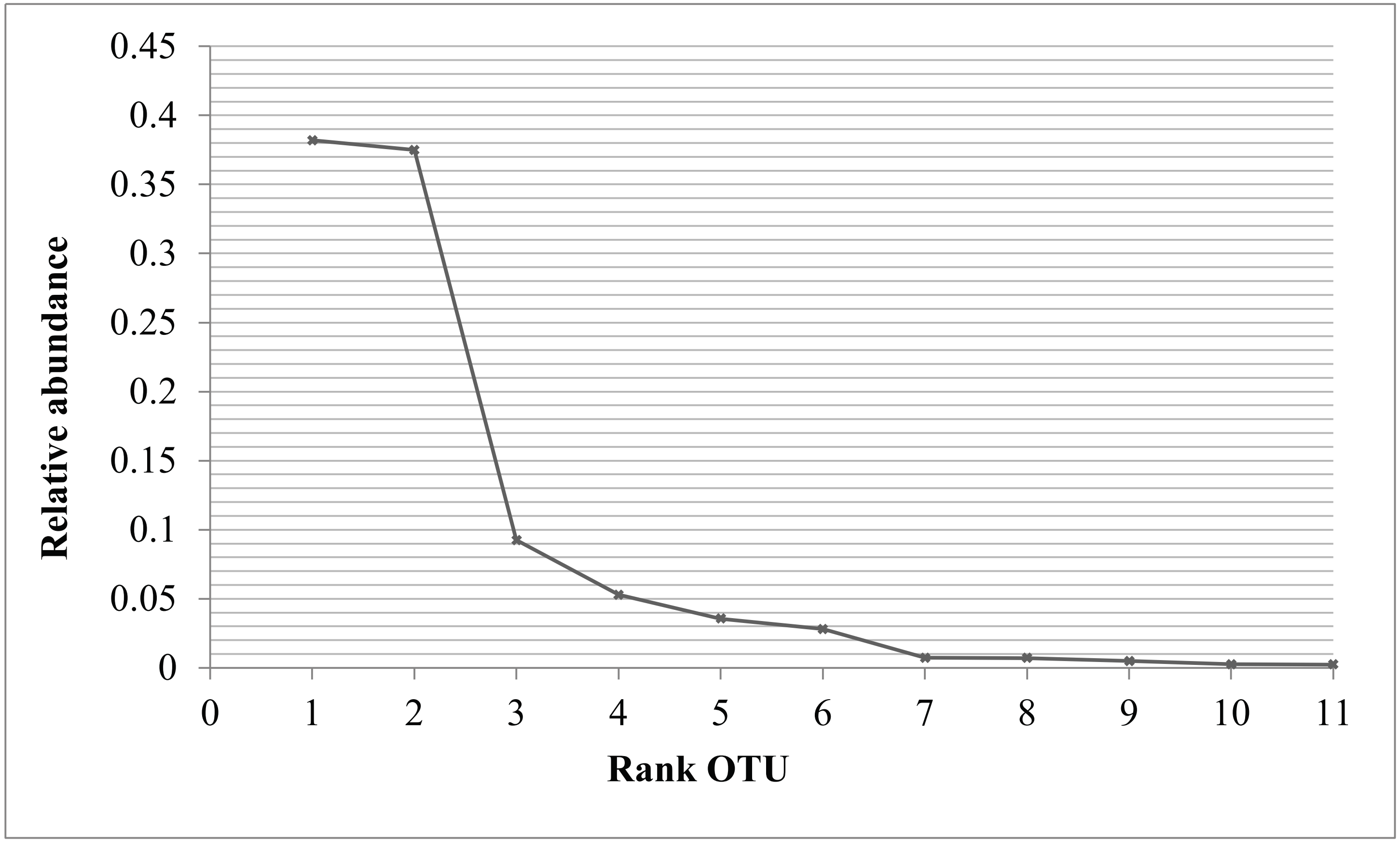

Fig. S2: OTU relative abundance from from Illumina sequencing.

Consensus sequences of bacterial components retrieved from mixed sequencing

Frischella perrara:
ATCGGATTGACTTGGGCGTAAAGGGCATGTAGTGTCGGATGATTAAGTTAGGTGTGAAAGCCCTCG
GGCTCAACCTGAGAATAGCATTTAAAACTGGTGATCTGGAGTACTGTAGAGGGAGGTGAGAATTCC
ACGTGTAGCGGTGAAATGCGTAGATGATGTGGAGGAACTACCGGTGGCGA

Gilliamella apicola:
ATCGGAATGACTGGGCGTAAAGGGCATGTAGGCGGATAATTAAGTTAGGTGTGAAAGCCCTGGGC
TCAACCTAGGAATTGCACTTAAAACTGGTTAACTAGAGTATTGGTAGAGGAAGGTAGAATTCCACG
TGTAGCGGTGAAATGCGTAGAGATGTGGAGGAATACCGGTGGCGA

Snodgrassella alvi:
TATCGGATACTTACTTGGGGCGTAAAGCGAGCGCAGATCTGGTTAATTAAGTCAGATGTGAAATCC
CCGAGCTCAACTTGGGGACGTGCATTTGAAACTGGTTAACTAGAGTGTGTCAGAGGAGAGGTAGA
ATTCCACGTGTAGCAGTGAAATGCGTAGAGATGTGGAGGAATCACCGATGGCGAA

Enterobacteriaceae:
ATCGGATTACTTGGGGCGTAAAGCGCACGRAGGCGGTRTGTCTAAGTCAGGATGTGAAATCCCCTG
GGCTCAACTCCTGGGAACMGCATTTCGAAACTGGCATGGACTAGAGTCTTGGTAGAGGGGGGTAG
GAATTCCAGGTGTAGCGGTGAAATGCGTAGATGATCTGYAGGAACTACCGGTGGCGA

Acetobacteraceae:
TGCGTGCGGGAAGTGACGTGGGGCGTAAAGGGCGCTGCTAGGCGTGTTTAGTACAGTCAGATGTG
AAATTCCCCTGGGGCTTAACCTGGGAGAGCTGCATTTGATACGTTAGCTAGAGACTAGAGTCGCGA
AAGAGGGTTGTGGAATCTCCCAGTGTAGAGGTGAAATTCGTAGATATTTGGGAAGAACACCGGTGT
GCGA

Rhizobiales bacterium:
GTTCGGATTTATCTGGGCGTAAAGCGCACGTAGGCGGATATTTAAGTCAGGGGTGAAATCCCGGGG
CTCAACCCCGGAACTTGCCTTTGATACTGGATATCTTGAGTATGGAAGAGGTAAGTGGAATTCCGA
GTGTAGAGGTGAAATTCGTAGATATTCGGAGGAACACCAGTGGCGA

Lactobacillus:
GTTCCGGGATTTATTGGGGCGTAAAGCGGACATCGCAGGCGGGWAGTAWTAAGTCAGCATGTGAA
AGCTCCRCAGGCCTTAACTCGTGGGAGASCTGCAGTCTTGAAACTAGTGTTTTCTTGAGTGCAGAA
AGGAGGAGTGGAACTCCATGTGTAGCGGTGGAAATGTCGTAGATATATGGAAGAAACACCAGGTG
GGGCAA

Fig. S3: Consensus sequences from bacterial components derived from mixed sequencing.

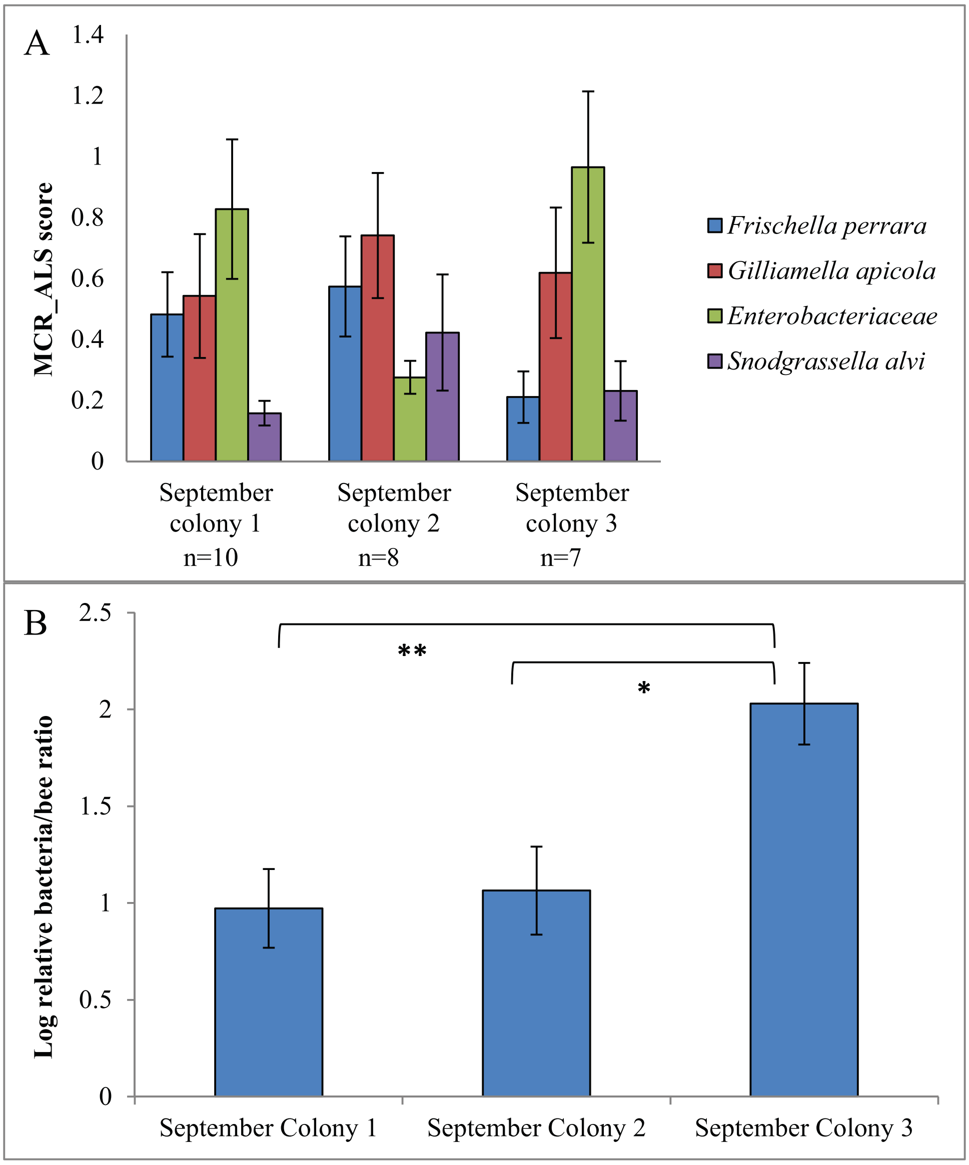

Fig. S4: Midgut/pyloric microbiota composition in September colonies. A) The MCR-ALS score, determined by mixed sequencing, represent approximately relative bacterial composition for the four dominating components in the honey bee midgut/pylorus without closure of the system. B) Mean relative bacteria quantity for September colonies. Calculated relative ratio between 16S rRNA genes and vitellogenin genes (bacteria/bee), in the midgut/pylorus determined by quantitative PCR, is shown. Markings show error bars of calculated SEM (68.2% CI) for each bacterium in each colony and T-test values with significant difference is shown; *=$p<0.05$, **=$p<0.01$. Shown is *n*; number of bees included in the final analyses each month.

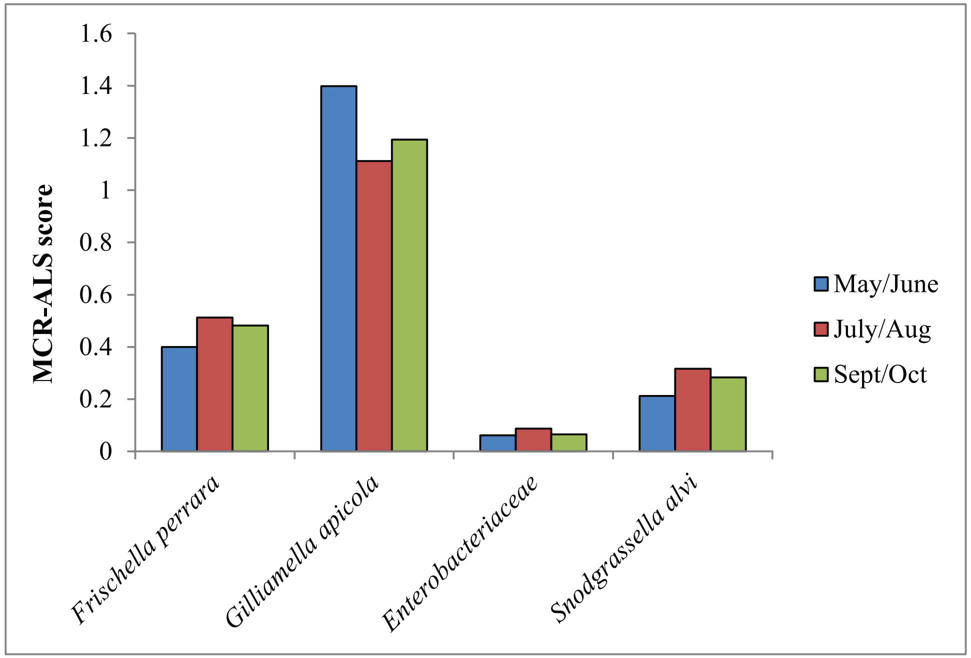

Fig. S5: Technical variation in reference to plate-to-plate variation in the mixed sequencing. The four components found in the Seasonal dataset were analyzed in DNA from one pooled control sample of honey bee midgut/pyorus (collected in July, *n*=10), which was extracted in three turns/on tree plates, in replicate on each plate, as indicated on the figure (May/June), (July/Aug), and (Sept/Oct). Graphed values are a calculated average value of two replicates for each extraction/time point after MCR-ALS analysis. The MCR-ALS score, determined by mixed sequencing, represent approximately relative bacterial composition in the honey bee midgut/pylorus without assuming closure of the system.
